# Deep learning-based denoising image reconstruction of body magnetic resonance imaging in children

**DOI:** 10.1007/s00247-025-06230-5

**Published:** 2025-04-05

**Authors:** Vanda Pocepcova, Michael Zellner, Fraser Callaghan, Xinzeng Wang, Maelene Lohezic, Julia Geiger, Christian Johannes Kellenberger

**Affiliations:** 1 Department of Diagnostic Imaging, University Children’s Hospital Zurich, Lenggstrasse 30, 8008 Zurich, Switzerland; 2 Children’s Research Center, University Children’s Hospital Zurich, Zurich, Switzerland; 3 Center for MR Research, University Children’s Hospital Zurich, Zurich, Switzerland; 4 GE Healthcare, Houston, TX USA; 5 GE Healthcare, Zurich, Switzerland

**Keywords:** Deep learning, Image reconstruction, Lung, Magnetic resonance imaging, Paediatric, Upper abdomen

## Abstract

**Background:**

Radial *k*-space sampling is widely employed in paediatric magnetic resonance imaging (MRI) to mitigate motion and aliasing artefacts. Artificial intelligence (AI)-based image reconstruction has been developed to enhance image quality and accelerate acquisition time.

**Objective:**

To assess image quality of deep learning (DL)-based denoising image reconstruction of body MRI in children.

**Materials and methods:**

Children who underwent thoraco-abdominal MRI employing radial *k*-space filling technique (PROPELLER) with conventional and DL-based image reconstruction between April 2022 and January 2023 were eligible for this retrospective study. Only cases with previous MRI including comparable PROPELLER sequences with conventional image reconstruction were selected. Image quality was compared between DL-reconstructed axial T1-weighted and T2-weighted images and conventionally reconstructed images from the same PROPELLER acquisition. Quantitative image quality was assessed by signal-to-noise ratio (SNR) and contrast-to-noise ratio (CNR) of the liver and spleen. Qualitative image quality was evaluated by three observers using a 4-point Likert scale and included presence of noise, motion artefact, depiction of peripheral lung vessels and subsegmental bronchi at the lung bases, sharpness of abdominal organ borders, and visibility of liver and spleen vessels. Image quality was compared with the Wilcoxon signed-rank test. Scan time length was compared to prior MRI obtained with conventional image reconstruction.

**Results:**

In 21 children (median age 7 years, range 1.5 years to 15.8 years) included, the SNR and CNR of the liver and spleen on T1-weighted and T2-weighted images were significantly higher with DL-reconstruction (*P*<0.001) than with conventional reconstruction. The DL-reconstructed images showed higher overall image quality, with improved delineation of the peripheral vessels and the subsegmental bronchi in the lung bases, sharper abdominal organ margins and increased visibility of the peripheral vessels in the liver and spleen. Not respiratory-gated DL-reconstructed T1-weighted images demonstrated more pronounced respiratory motion artefacts in comparison to conventional reconstruction (*P=*0.015), while there was no difference for the respiratory-gated T2-weighted images. The median scan time per slice was reduced from 6.3 s (interquartile range, 4.2 – 7.0 s) to 4.8 s (interquartile range, 4.4 – 4.9 s) for the T1-weighted images and from 5.6 s (interquartile range, 5.4 – 5.9 s) to 4.2 s (interquartile range, 3.9 – 4.8 s) for the T2-weighted images.

**Conclusion:**

DL-based denoising image reconstruction of paediatric body MRI sequences employing radial *k*-space sampling allowed for improved overall image quality at shorter scan times. Respiratory motion artefacts were more pronounced on ungated T1-weighted images.

**Graphical Abstract:**

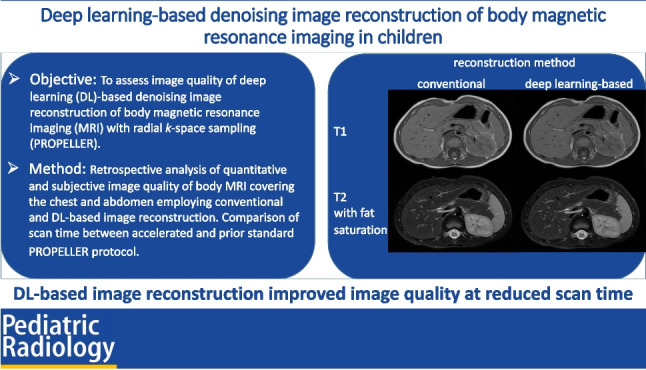

## Introduction

Magnetic resonance imaging (MRI) is an important cross-sectional imaging method in diverse clinical settings of the paediatric population due to its superior resolution, tissue contrast, and lack of ionising radiation in comparison to computed tomography. Artefacts in paediatric MRI represent a big challenge for achieving diagnostic image quality. Motion artefacts are usually caused by patients’ gross motion, respiratory motion, vascular pulsation, bowel peristalsis, or cardiac contraction [1]. Several approaches to minimise or curtail motion artefacts, such as suppressing signal from moving tissues, physiologic gating, swapping frequency- and phase-encoding directions, use of ultrafast sequences, parallel imaging, or rotating *k*-space filling pattern, have been developed to improve the image quality and thus increase the diagnostic certainty [2]. Radial *k*-space sampling, alongside the standard Cartesian data acquisition method, is one of the original image reconstruction approaches since the introduction of MRI in medical imaging in the 1980s [3]. It gained importance in the last two decades and has been increasingly implemented as a standard part of the imaging protocols in paediatric MRI due to its motion artefact suppression and increased image quality [4, 5]. Since the introduction in 1999 by Pipe [6], the periodically rotated overlapping parallel lines with enhanced reconstruction (PROPELLER) method has been well established in imaging the head, lung, abdomen, or shoulder as a result of its superior anatomical detail depiction [5, 7–10]. However, this acquisition technique increases the scanning time by the factor of *π*/2 in comparison to conventional Cartesian sequences due to oversampling of the central *k*-space. Since then, approaches to address this limitation have been investigated. Tamhane et al. proposed a combination of under-sampling of the *k*-space by reducing the number of blades and iterative reconstruction to shorten the acquisition time while preserving the motion correction, nevertheless reducing the signal-to-noise ratio [11], which is already low in body MRI. Recent advances in computer science have enabled broad application of artificial intelligence (AI) tools in medical imaging. Deep learning (DL) as a subset of AI has been recently implemented in various areas of diagnostic imaging from image reconstruction to lesion detection [12]. DL can be employed in extrapolation of unknown signals from existing ones in under-sampled *k*-space or can be applied in different signal-processing steps of MR image reconstruction, thus improving the image quality and shortening the examination time [13, 14]. Denoising is one of the image reconstruction solutions enhancing either raw *k*-space or image domain data and its clinical value has been recently demonstrated in various studies [15–18].

In this work, we evaluate the image quality and compare the scan time length of conventional and AI-based denoising image reconstruction of T1-weighted and T2-weighted PROPELLER sequences in body MRI in children.

## Materials and methods

### Patients

Ethical approval was obtained from the local ethical committee (BASEC-Nr. 2023-00703) for this retrospective study. Consent was obtained for evaluation of existing imaging data and the HIPAA privacy rules were applied. Eighty consecutive patients who underwent thoraco-abdominal MRI on a 1.5-T scanner (Signa Artist, GE HealthCare, Waukesha, WI) at our institution employing PROPELLER sequences with DL-based image reconstruction were considered for this study. The inclusion criterion was the availability of a previous thoraco-abdominal MRI examination with conventionally reconstructed PROPELLER sequences to enable comparison of scan time between the previous PROPELLER protocol and the current accelerated PROPELLER protocol.

### Imaging protocol and post-processing

The patients underwent thoraco-abdominal imaging with multichannel surface coils (AIR™ Coil, GE HealthCare, Waukesha, WI) covering the chest and abdomen. The routine protocol included a 3-dimensional (D) short tau inversion recovery fast spin echo sequence (CUBE) and diffusion-weighted imaging, axial and coronal T1-weighted PROPELLER sequences, and respiratory-gated axial T2-weighted PROPELLER with fat saturation. After intravenous injection of a single dose of gadolinium-based contrast agent (gadoteric acid, Dotarem, Guerbet AG, Zürich, Switzerland), a dynamic 3-D acquisition (DISCO) was followed by a 3-D T1-weighted fast gradient echo sequence (LAVA).

Subjects of this study were the T1-weighted and T2-weighted axial PROPELLER images obtained before contrast administration. The PROPELLER sequences in this study were acquired with higher image matrix and less signal averages than in our previous standard protocol (scan parameters are given in Table 1). Scan time was extracted from MRI scanner internal timing, which is saved to digital imaging and communications in medicine (DICOM) meta data, and is reported here as seconds per slice for normalisation. By the time of the study, the DL-based image reconstruction algorithm (AIR™ Recon DL, GE HealthCare, Waukesha, WI) was provided by the vendor as a research option for paediatric body imaging. The reconstruction pipeline includes a residual neural network that was trained to reduce the noise and truncation artefacts and improve edge sharpness from the raw blade-combined images. The convolutional residual network was trained with a supervised learning approach using pairs of near-perfect, high-resolution, and conventional images [15, 19, 20]. The model was integrated into the conventional PROPELLER reconstruction pipeline, generating conventional and DL-reconstruction images from the same acquisition. The impact of DL-reconstruction on the image quality in comparison to the conventional approach was evaluated. Scan times from the current PROPELLER acquisitions were compared to those from a prior examination acquired with our old standard PROPELLER protocol.

**Table 1:** Technical settings of standard and accelerated PROPELLER sequences for paediatric body magnetic resonance imaging

	Standard protocol		Accelerated protocol	
	T1-weighted	T2-weighted	T1-weighted	T2-weighted
TR (ms)	731; 458 – 900	3,529; 2,308 – 13,846	793; 500 – 898	3,158; 2,143 – 5,455
TE (ms)	25; 17 – 27	93; 88 – 103	22; 21 – 26	98; 41 – 103
Slice thickness (mm)	5; 3 – 5	5; 3 – 5	5; 4 – 8	5; 4 – 8
Echo train length	8; 6 – 8	28; 28 – 30	8; 8 – 8	32; 28 – 32
Matrix	256^2^ – 352^2^	256^2^ – 320^2^	280^2^ – 420^2^	280^2^ – 400^2^
FOV (mm)	200 – 340	200 – 340	200 – 300	200 – 300
Flip angle (°)	90	160	90	160
NEX	2.5; 2 – 2.6	2.2; 2 – 2.6	1.7; 1.5 – 3	1.7; 1.6 – 3.1
Number of slices	48; 27 – 78	33; 24 – 60	58; 31 – 78	36; 9 – 61
Respiratory gating	No	Yes	No	Yes

Data are given as range, or median; range

*FOV* field of view, *NEX* number of excitations, *TE* echo time, *TR* repetition time

### Quantitative image assessment

Signal-to-noise ratio (SNR) of the liver and spleen and contrast-to-noise ratio (CNR) of the liver and spleen in comparison to spinal musculature were calculated to assess quantitative image quality.

SNR is calculated as the ratio of signal intensity (SI) of the organ parenchyma to the standard deviation (SD) of the signal intensity of the background – SD air. CNR is defined as the difference in signal contribution (SI) of the tissue of interest and a reference tissue, divided by SD air.

$$\mathrm{SNR}=\frac{\mathrm{SI}\;\left(\mathrm{liver}/\mathrm{spleen}\right)}{\mathrm{SD}\left(\mathrm{air}\right)}\quad \mathrm{CNR}=\frac{\mathrm{SI}\;\left(\mathrm{liver}/\mathrm{spleen}\right)-\mathrm{SI}\;\left(\mathrm{muscle}\right)}{\mathrm{SD}\;\left(\mathrm{air}\right)}$$

**Fig. 1:**
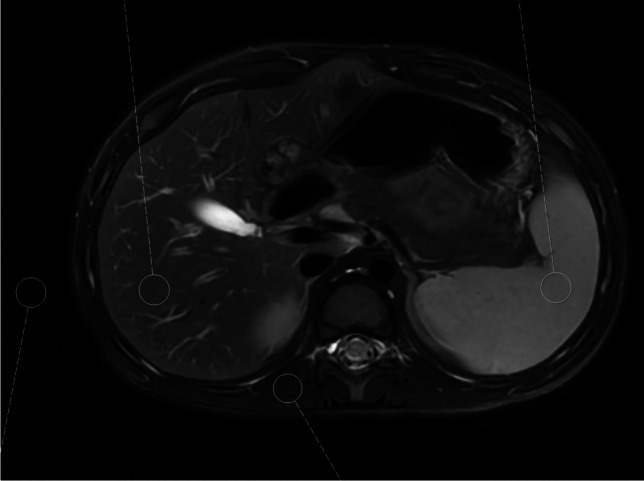
Example of region of interest placement on an axial deep learning-reconstructed T2-weighted PROPELLER image before contrast agent administration to determine signal-to-noise ratio and contrast-to-noise ratio

Measurements were made within circular regions of interest (ROI) of diameters ranging from 9.1 mm to 10.6 mm (area ranging from 65 mm^2^ to 89 mm^2^) within the liver and spleen parenchyma omitting the vasculature and within the air next to the body. If technically possible, the ROIs in the liver, spleen, and air were positioned in the same line on the *x*-axis. To determine the CNR, autochthonous spinal muscles were chosen as tissue of reference. The ROI was positioned at the identical location of the organ, muscle, and air on the T1-weighted and T2-weighted images. The example of ROI placement is given in Fig. 1.

### Qualitative image assessment

Prior to the data analysis, the images with conventional and DL-reconstruction were anonymised, and the sequence information was removed. The images were independently reviewed by three board-certified paediatric radiologists (C.J.K. with 30 years, V.P. with 13 years, and M.Z. with 8 years of experience) in random order, and the observers were blinded to the patient clinical data. The images were evaluated for the presence of noise, motion artefact, visibility of the peripheral lung vessels and subsegmental bronchi at the lung bases, abdominal organ margins, and visibility of the peripheral liver and spleen vessels using a 4-point Likert scale.

The degree of noise was scored with 3=no noise, 2=mild noise, 1=noise potentially obscuring the vessels, and 0=not diagnostic. The motion artefact was evaluated as follows: 3=no artefact, 2=mild, 1=moderate, 0=severe. The observers assessed the visibility of peripheral lung vessels (1 cm subpleural), subsegmental bronchi and peripheral liver, and spleen vessels in a following manner: 3=well seen with sharp margins, 2=well seen, blurred, 1=hardly visualised, 0=not visualised. Margins of the abdominal organs were scored with 3=well seen with sharp margins, 2=well seen, blurred, 1=hardly visualised, and 0=not visualised.

For each sequence and corresponding reconstruction method, six image quality items were evaluated by three observers and used for the cumulative image quality score. The median of the cumulative image quality score from three readers was used for comparison between reconstruction types.

### Statistics

The primary analysis showed that the data does not follow a normal distribution. All data collected for the quantitative and the qualitative image assessment were compared using the Wilcoxon signed-rank test. A statistical significance of *P*-value <0.05 among the reconstruction methods for each sequence has been determined. The reliability of agreement for image quality between the three readers was determined by interclass correlation coefficient (ICC). ICC values of <0.5 indicated poor, between 0.5 and <0.75 moderate, between 0.75 and <0.9 good, and between 0.9 and 1 excellent agreement. Statistical analysis was conducted using the software XLSTAT, version 2023.1.1 (Lumivero (2025); XLSTAT statistical and data analysis solution, New York, NY; https://www.xlstat.com/en) and MedCalc® Statistical Software version 23.1.6 (MedCalc Software Ltd, Ostend, Belgium; https://www.medcalc.org; 2025).

## Results

MRI scans of 21 children (11 males and 10 females) with median age 7 years (ranging from 1.5 years to 15.8 years), who met the inclusion criteria, were investigated in this study. The clinical indication for chest and abdominal MRI at least including the lung bases was follow-up of malignant tumours in 19 cases, including neuroblastoma, nephroblastoma, hepatoblastoma, and T-lymphoblastic or Burkitt lymphoma. Two cases were referred for a follow-up examination of congenital haemangioma of the liver and echinococcosis of the lung and the liver, respectively. Nineteen patients were scanned while sedated and two patients did not require any sedation. Not all examinations were performed with contrast medium administration.

### Quantitative image quality

**Fig. 2:**
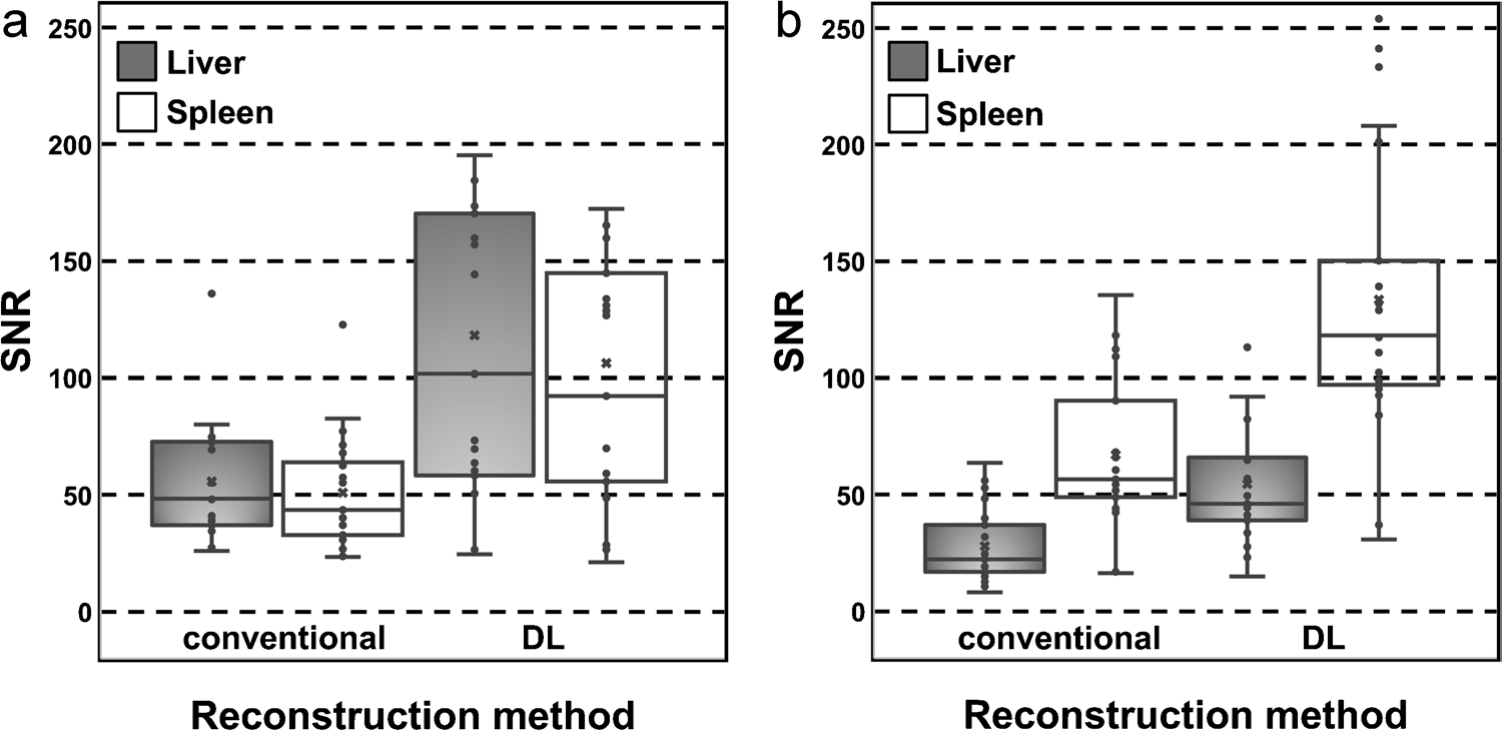
**a**, **b** Box plots showing signal-to-noise ratio of the liver and spleen in conventional and deep learning-based reconstruction of T1- (**a**) and T2- (**b**) weighted magnetic resonance images employing radial *k-*space sampling (PROPELLER)

**Fig. 3:**
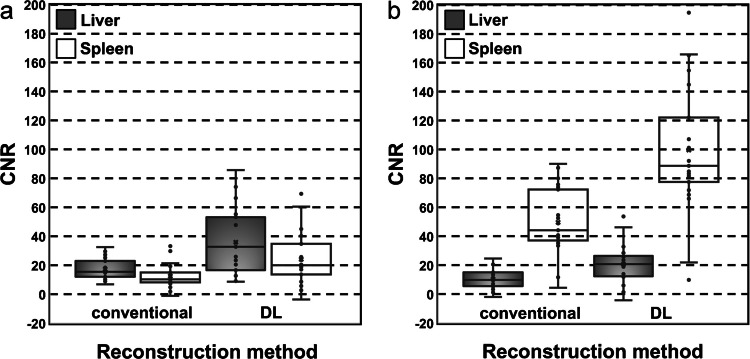
**a**, **b** Box plots showing contrast-to-noise ratio of the liver and spleen in conventional and deep learning-based reconstruction of T1- (**a**) and T2- (**b**) weighted magnetic resonance images employing radial *k*-space sampling (PROPELLER)

For DL-reconstructed T1-weighted sequences, the median SNR of the liver was 101.6 (interquartile range, 58.2 – 170.4) and of the spleen 92.3 (interquartile range, 55.7 – 144.7); the median CNR of the liver was 32.9 (interquartile range, 16.7 – 53.1) and of the spleen 20.1 (interquartile range, 13.6 – 34.8). For the conventionally reconstructed T1-weighted images, the median SNR of the liver was 48.4 (interquartile range, 37.0 – 72.7) and of the spleen 43.5 (interquartile range, 32.8 – 64.0); the median CNR of the liver was 15.4 (interquartile range, 12.1 – 23.0) and of the spleen 10.3 (interquartile range, 8.2 – 15.0). SNR and CNR of the liver and spleen in DL-reconstructed sequences were significantly higher (*P<*0.001 respectively) than of images reconstructed by the conventional method (Figs. 2a and 3a).

For DL-reconstructed T2-weighted images, the median SNR of liver was 46.0 (interquartile range, 38.9 – 65.9) and of the spleen 118.1 (interquartile range, 97.0 – 150.3); the median CNR of the liver was 20.8 (interquartile range, 12.2 – 26.5) and of the spleen 88.7 (interquartile range, 77.5 – 122.2). For the conventionally reconstructed T2-weighted images, the median SNR of the liver was 22.2 (interquartile range, 17.0 – 36.9) and of the spleen 56.6 (interquartile range, 49.0 – 90.3); the median CNR of the liver was 9.8 (interquartile range, 5.6 – 15.0) and of the spleen 44.2 (interquartile range, 37.0 – 72.3). SNR and CNR of the liver and spleen in images reconstructed by the DL-based algorithm were significantly higher (*P<*0.001 respectively) than in conventionally reconstructed sequences (Figs. 2b and 3b).

### Qualitative image quality

**Fig. 4:**
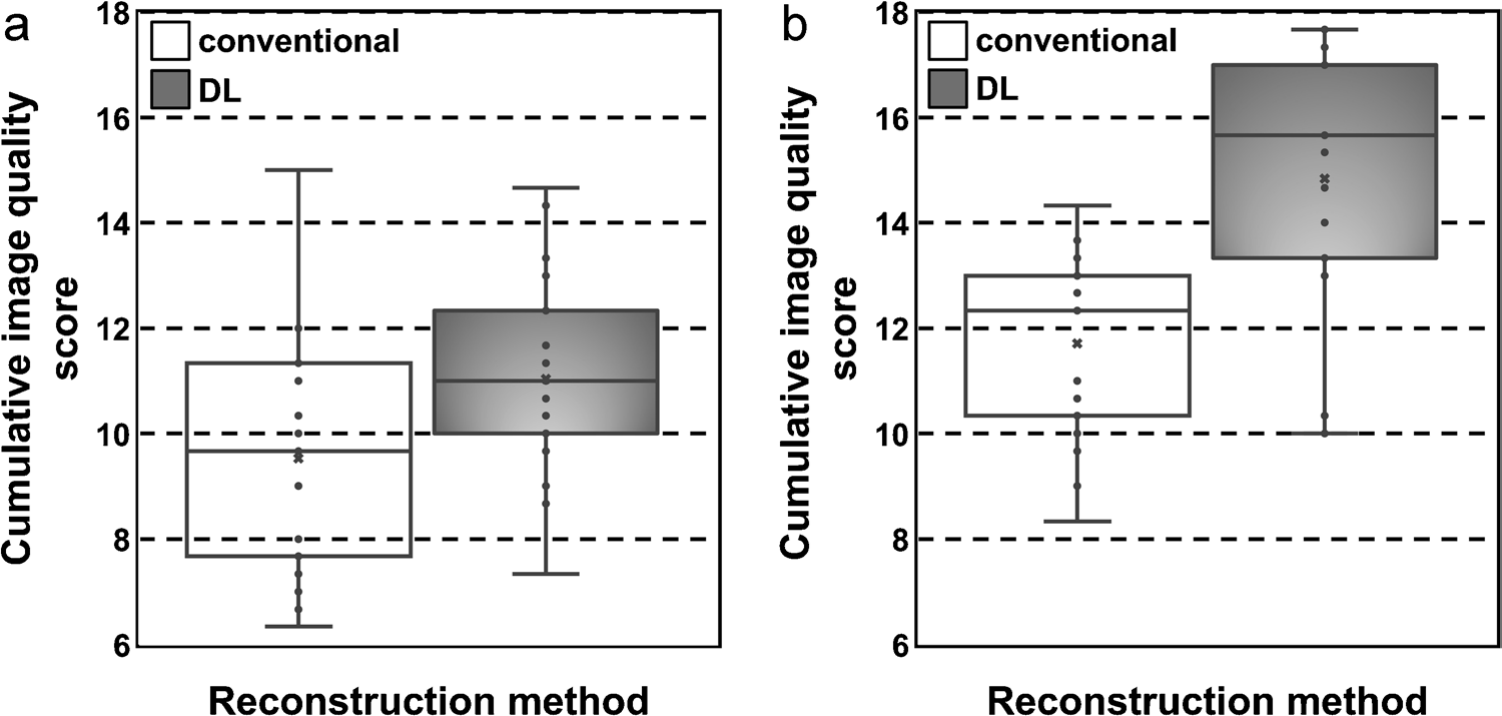
**a**, **b** Box plots showing cumulative image quality score in conventional and deep learning-based reconstruction of T1- (**a**) and T2- (**b**) weighted magnetic resonance images employing radial *k*-space sampling (PROPELLER)

The details of the subjective image quality comparison are given in Table 2. The DL-reconstructed images showed higher cumulative image quality score (*P*<0.002 for T1-weighted, *P*<0.001 for T2-weighted) and less apparent noise (*P<*0.005 for T1-weighted, *P*<0.001 for T2-weighted) than those obtained by the conventional reconstruction method (Fig. 4). Peripheral vessels in the lung, liver, and spleen, subsegmental bronchi at the lung bases, and the abdominal organ margins were better delineated in the DL-reconstructed images than in the conventional T1-weighted images. The peripheral vessels of the lung bases, the subsegmental bronchi, abdominal organ margins, and peripheral vessels of the liver and spleen were significantly better delineated in the DL-reconstructed T2-weighted images than in the sequences obtained with conventional reconstruction. The respiratory motion artefact on the T1-weighted sequences appeared more pronounced on DL-reconstructed images than with conventional reconstruction (*P=*0.015). There was no statistically significant difference of motion artefact presentation in the T2-weighted DL-reconstructed images in comparison to the conventionally reconstructed ones (*P=*0.664).

**Table 2:** Subjective image quality of PROPELLER sequences with conventional and deep learning-based reconstruction for paediatric body magnetic resonance imaging assessed by 3 readers

	Conventional reconstruction	DL-based reconstruction	*P*-value
T1-weighted sequences			
Cumulative image quality score (0–18)	9.67; 7.67 – 11.33	11; 10 – 12.33	0.002
Noise (0–3)	2; 1.67 – 2	2.67; 2.33 – 2.67	0.005
Respiratory motion artefact (0–3)	1.67; 1.33 – 2.33	1.33; 1 – 2	0.015
Lung vessels (0–3)	1.67; 1.33 – 2	2; 1.67 – 2	0.005
Subsegmental bronchi (0–3)	1.67; 1 – 1.67	1.67; 1.33 – 2	0.006
Abdominal organ margins (0–3)	1.33; 1.33 – 1.67	2; 1.67 – 2	0.003
Peripheral vessels in the liver and spleen (0–3)	1.33; 1 – 1.67	1.33; 1.33 – 2	0.027
Scan time (s/slice)	6.3; 4.2 – 7	4.8; 4.4 – 4.9	
T2-weighted sequences			
Cumulative image quality score (0–18)	12.33; 10.33 – 13	15.67; 13.33 – 17	<0.001
Noise (0–3)	2; 1.67 – 2	3; 2.33 – 3	<0.001
Respiratory motion artefact (0–3)	2.67; 2.33 – 3	2.67; 2.33 – 3	0.664
Lung vessels (0–3)	2; 1.67 – 2	2.67; 2 – 3	<0.001
Subsegmental bronchi (0–3)	2; 1.33 – 2	2.33; 2 – 2.67	<0.001
Abdominal organ margins (0–3)	1.67; 1.33 – 2	2.33; 1.67 – 3	<0.001
Peripheral vessels in liver and spleen (0–3)	2; 1.67 – 2	2.67, 2.33 – 2.67	<0.001
Scan time (s/slice)	5.6; 5.4 – 5.9	4.2; 3.9 – 4.8	

Data are given as median; interquartile range

The cumulative image quality score of the DL-reconstructed and accelerated PROPELLER sequences was higher than that of the conventionally reconstructed images from the prior examination, with a median difference of 1.7 for T1-weighted images (*P=*0.003) and 3.7 for T2-weighted images (*P<*0.001).

**Fig. 5:**
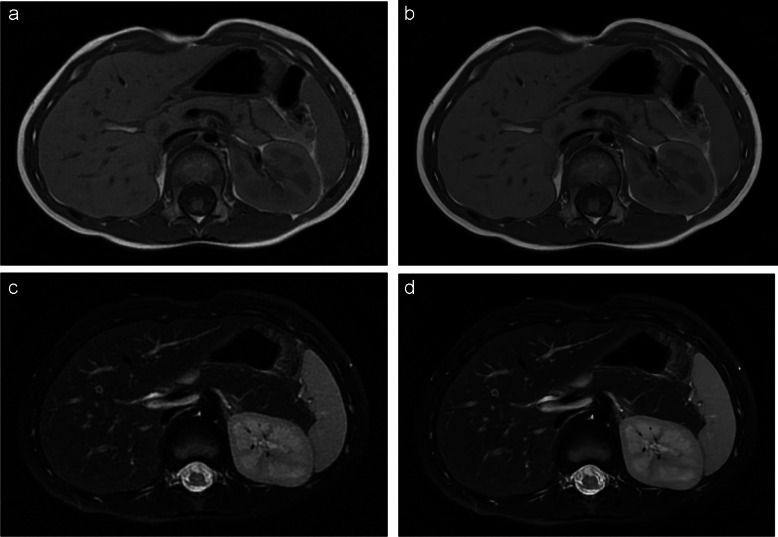
**a**-**d** T1- (**a**, **b**) and T2- (**c**, **d**) weighted axial PROPELLER images of the upper abdomen before administration of contrast agent demonstrating reduced noise, sharper delineation of liver and spleen vessels, and better depiction of organ margins with deep learning-based reconstruction (**b**, **d**) than with conventional reconstruction (**a**, **c**)

**Fig. 6:**
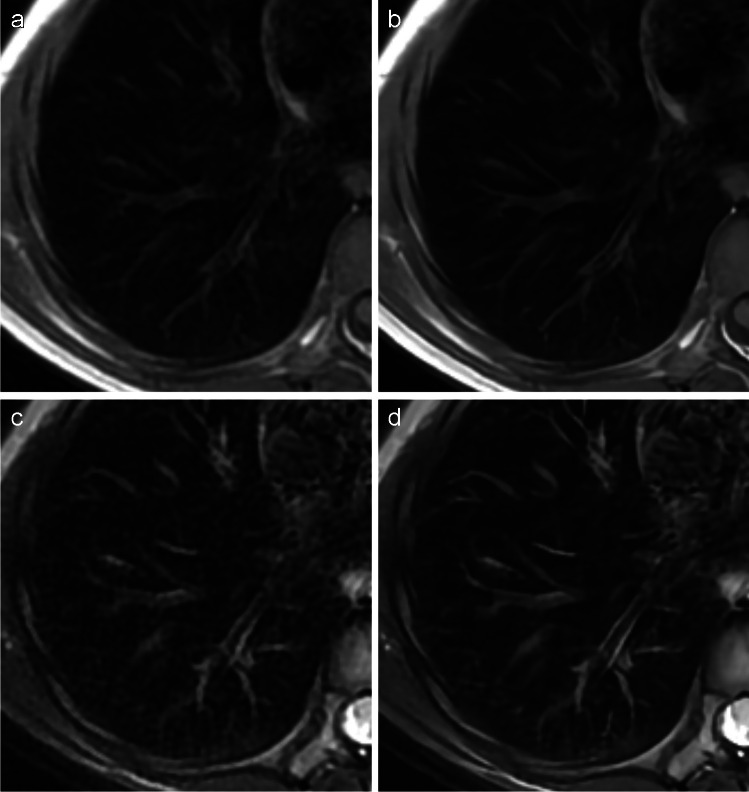
**a-d** Details of T1- (**a**, **b**) and T2- (**c**, **d**) weighted axial PROPELLER images of the basal lung without contrast agent administration demonstrating sharper delineation of subsegmental bronchi and less noise in deep learning-based reconstruction (**b**, **d**) than in conventional reconstruction (**a**, **c**)

Examples of conventionally and DL-reconstructed T1-weighted and T2-weighted images of the basal lung and abdominal organs are shown in Figs. 5 and 6.

### Inter-rater agreement

There was moderate inter-observer agreement for the individual cumulative image quality scores of the three readers (absolute agreement of single measures, ICC 0.71, 95% confidence interval 0.61 – 0.79). In order to improve reliability of the image quality assessment, the median of the three individual cumulative image quality scores was used (absolute agreement of 3 ratings, ICC 0.88 (95% confidence interval 0.82 – 0.92)).

### Scan time

Compared to the prior MRI examination obtained with our old standard PROPELLER protocol, the median scan time per slice was reduced from 6.3 s (interquartile range, 4.2 – 7.0 s) to 4.8 s (interquartile range, 4.4 – 4.9 s) for the T1-weighted images and from 5.6 s (interquartile range, 5.4 – 5.9 s) to 4.2 s (interquartile range, 3.9 – 4.8 s) for the T2-weighted images. DL-reconstruction ran automatically in the background following conventional image reconstruction and did not interfere with or increase overall examination acquisition.

## Discussion

In this study, we show that AI-based denoising image reconstruction can improve image quality at reduced scanning time of body MRI in the paediatric population. In an accelerated PROPELLER protocol with 25% shortened scan time, the quality of the DL-reconstructed images was better than that of the conventionally reconstructed images. This allows to decrease the total imaging time in less cooperative patients or shorten the sedation time in young children.

Consistent with previous research [16–18, 21–24], both T1-weighted and T2-weighted DL-reconstructed PROPELLER images showed improved quantitative and subjective image quality. The SNR and CNR were higher, and the images appeared less noisy. In the lungs, the peripheral vessels and subsegmental bronchi were more clearly depicted. The solid abdominal organs showed sharper borders and were better delineated from surrounding tissues. The peripheral vessels in the liver and spleen were more clearly seen. The overall subjective image quality was higher for T2-weighted than for T1-weighted DL-reconstructed images and the difference of subjective image quality between conventionally and DL-reconstructed T1-weighted images was smaller than that for T2-weighted images, which may be explained by the use of respiratory gating for T2-weighted images but not for T1-weighted images. Of the rated image quality items, all were improved with DL-reconstruction except for the respiratory motion artefacts. In order to minimise respiratory motion artefacts, we utilised respiratory gating for the T2-weighted acquisitions, which is not possible with T1-weighted spin echo or PROPELLER acquisitions due to the length of the repetition time (TR) around 500 ms not fitting in the interval between breaths. With conventional reconstruction, the respiratory-gated T2-weighted images revealed less respiratory motion artefacts than the non-gated T1-weighted images.

On the respiratory-gated T2-weighted images, the respiratory motion artefacts were not significantly different between the conventional and DL-reconstruction. However, on the T1-weighted images, the respiratory motion artefacts were more conspicuous with DL-reconstruction than with conventional image reconstruction but still not deleterious. It is well known from Cartesian fast spin echo acquisitions that DL-reconstruction motion artefacts become more apparent due to the denoising performance of the algorithm [12, 15, 17]. Moreover, the more noticeable respiratory artefact in T1-weighted DL-reconstructed images causes larger intensities in air and results in higher interquartile range of SNR and CNR measurements. It is the respiratory artefact itself that reduces the image quality. Consequently, motion artefacts need to be minimised during sequence acquisition as it is difficult to correct non-rigid body motion in PROPELLER reconstruction.

With the increasing use of AI-based image reconstruction for MRI, issues such as the obscuring of anatomical structures or true lesions and addition of extra anatomical structures have been reported [25]. In our study, we did not observe any loss or addition of structures on the studied T1-weighted and T2-weighted images. On the contrary, small vessels were more clearly visualised.

The main limitation of this study is the small number of cases fulfilling the inclusion criteria. Nonetheless, we show that DL-based image reconstruction significantly improves image quality. Another limitation is that the examinations were only performed on 1.5 T; and therefore, our results cannot be generalised to other field strengths. While our study population comprised patients undergoing regular MRI follow-up for identification of a relapse and metastases, note that we did not analyse the ability of DL-reconstructed MRI sequences for organ lesion detection since the number of residual tumours or other organ lesions was too low to scrutinise.

## Conclusion

DL-based denoising image reconstruction in paediatric body MRI employing radial *k*-space sampling improves the image quality by reduction of noise, increase of contrast, and sharper visualisation of organ structures. The improved image quality allows for shorter scan times. Respiratory motion artefacts may become more pronounced with DL-based denoising reconstruction and therefore need to be minimised at image acquisition.

## Data Availability

Images (Figs. 1, 5 and 6) were extracted from clinical picture archiving and communication system (PACS) and are not publicly available in order to protect patient privacy. Data supporting Table 1 are stored in PACS/ digital imaging and communications in medicine (DICOM). Data supporting Figs. 2 and 3 were measured in clinical PACS (not publicly available) and analysed in XLSTAT. Data supporting Fig. 4 and Table 2 were obtained through image evaluation in clinical PACS and analysed in XLSTAT.
